# Drug-coated Balloons for Small Coronary Disease—A Literature Review

**DOI:** 10.1007/s11886-021-01586-0

**Published:** 2021-10-14

**Authors:** Ketina Arslani, Raban Jeger

**Affiliations:** Department of Cardiology, University Hospital Basel, University of Basel, Petersgraben 4, CH-4031 Basel, Switzerland

**Keywords:** Coronary artery disease, Small vessel disease, Drug-coated balloon, Drug-eluting balloon, Percutaneous coronary intervention

## Abstract

**Purpose of Review:**

In the interventional treatment of coronary artery disease, new-generation drug-eluting stents (DES) currently are the standard treatment. In addition, drug-coated balloons (DCB) are a well-established option for the treatment of in-stent restenosis in both bare-metal stents (BMS) and DES, where DCBs deliver an antiproliferative drug without the necessity of re-implanting a stent. Since the field of use for DCB has increasingly been extended to other indications such as de novo lesions in small vessel disease (SVD), a review of literature may be useful.

**Recent Findings:**

Recent randomized trial data show good efficacy and safety for DCB in de novo lesions, especially in small coronary arteries, and confirm long-term clinical efficacy and safety up to three years.

**Summary:**

DCB are an attractive and safe option in the treatment of de novo lesions in SVD.

## Introduction

Percutaneous coronary intervention (PCI) is the treatment of choice for patients with acute or chronic coronary artery disease (CAD) [1]. In the early days of PCI, the use of plain old balloon angioplasty (POBA) enabled the percutaneous treatment of CAD. Since then, the technique has evolved to the use of bare-metal stents (BMS), first-generation drug-eluting stents (DES), and second-generation and newer generation DES [2–5] as POBA was limited by elastic recoil, dissection, and restenosis. The development of these technologies eventually led to an optimization of efficiency and safety. However, in certain anatomical subsets such as small vessel disease (SVD), the use of DES is still challenging and seems to be associated with suboptimal results such as an increased risk for in-stent restenosis (ISR) [6–10]. This constitutes a significant issue, as SVD is documented in up to 30% of patients with CAD undergoing PCI [11].

Drug-coated balloons (DCB) are an alternative treatment strategy for CAD based on the fast transfer of antiproliferative drugs into the vessel wall during balloon inflation and is an established option in patients with ISR [12, 13]. In de novo lesions in small coronary arteries, DCB has evolved to a valid alternative to DES as shown in several non-randomized trials and subsequently also in a large randomized multicenter study with similar rates of major adverse cardiac events (MACE) up to 3 years in patients treated either with DCB or second-generation DES [14••, 15•].

## Methods

After a detailed search of PubMed according to the established methods and in adherence to the Preferred Reporting Items for Systematic Review and Meta-Analyses (PRISMA) statement [16], this review includes only English language studies. The keywords used are as follows: “drug-eluting stents” OR “DES” OR “drug-eluting balloons” OR “drug-coated balloons” OR “DCB” OR “DEB”. Databases were screened up until 9 May 2021. The most recent and comprehensive data were used and, in addition, references from original and review articles were checked and included.

### Small Vessel Disease – Definition and Prevalence

**Fig. 1:**
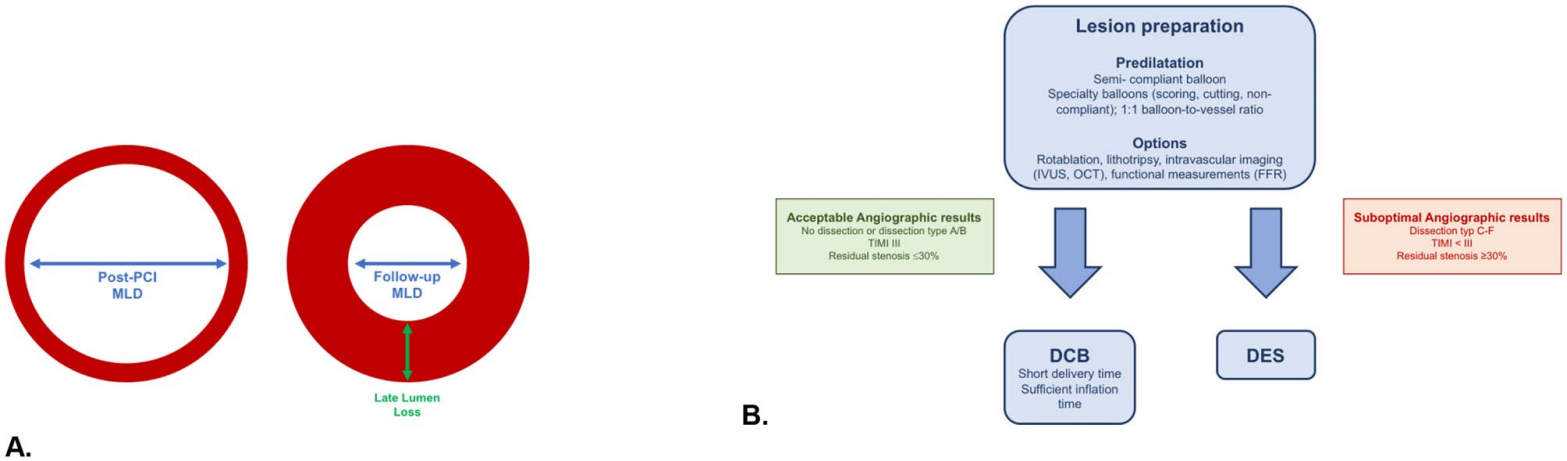
**A** Illustration of Late Lumen Loss and Minimum Lumen Diameter. **B** Drug-coated Balloon Strategy. Adapted from: Jeger RV et al. JACC Cardiovasc Interv. 2020;13(12):1391–402. https://doi.org/10.1016/j.jcin.2020.02.043, with permission from Elsevier) [17]. DCB: drug-coated balloon, DES: drug-eluting stent, FFR: fractional flow reserve, IVUS: intravascular ultrasound, OCT: optical coherence tomography, TIMI: thrombolysis in Myocardial infarction, MLD: Minimum lumen diameter, PCI: percutaneous coronary intervention

The International DCB Consensus Group suggested that a vessel of < 3 mm should be considered as “small”. SVD is therefore a lesion with a reference vessel diameter of < 3 mm [17]. SVD is fairly common and can be found in up to 30% of patients with symptomatic CAD. Specifically, patients with diabetes or chronic renal failure are more prone to develop SVD. Revascularization of SVD still represents a challenge due to the increased risk of adverse clinical events [8, 18]. Reference vessel diameter is defined as the average of diameter proximally and distally to the target lesion segment. A specific minimum lumen diameter (MLD) is aimed for when treating a vessel or lesion, which is based on the reference vessel diameter. MLD initially increases after the procedure and decreases at follow-up. Late lumen loss is defined as the difference between the postprocedural MLD and the MLD at follow-up (Fig. 1A). Preparation of the lesion and subsequent stent implantation leads to arterial injury and to neointimal hyperplasia. The quantity of neointimal hyperplasia is independent of the vessel size and thus the late lumen loss as an absolute number is similar in all vessel sizes [8]. Small vessels have a limited capacity to adapt to neointima formation without compromising blood flow after stent implantation and therefore restenosis occurs more often. It is natural that a minimal late lumen loss after treatment of SVD is crucial for optimal long-term results.

### Drug-coated Balloons – Technology

DCBs are semi-compliant balloons with a coating of an anti-proliferative drug. The treatment strategy entails that the drug is rapidly and homogenously transferred to the vessel wall from a lipophilic matrix during single balloon inflation. Therefore, the vessel is treated without leaving any residue behind. The following factors are essential for the successful use of a DCB: choice of DCB, lesion preparation, and correct application of the specific balloon. The lipophilic matrix ensures an undamaged transfer through the vascular system to the site of application and makes a fast and homogeneous transfer of the medication into the vascular wall possible after inflation. Various DCBs are available for the treatment of coronary vessels. In most DCB, paclitaxel is still the most widely used antiproliferative drug with a dose between 2 and 3.5 μg/mm^2^. This highly lipophilic drug is embedded in a specific coating matrix and has proven to be effective as it is a potent antiproliferative agent and chemically stable. Successful drug transfer is dependent on coating formulation and technique of the procedure. Among others, carrier excipients such as iopromide, urea and shellac have been investigated for optimal drug delivery (Table 1) [19]. There has been some contradictory data on possible increased mortality in patients treated with paclitaxel-coated balloons and stents [20, 21]. However, the finding of increased mortality in patients with peripheral arterial occlusive disease treated with paclitaxel-coated balloons [20, 21] is based on a meta-analysis with major methodological limitations that prevent a reliable interpretation, while a large meta-analysis in patients with coronary artery disease treated with paclitaxel-coated balloons did not find any evidence for increased mortality [20, 21]. Therefore, the use of paclitaxel-coated balloons in coronary artery disease may be considered as safe. A special feature of paclitaxel is the potential phenomenon of late positive remodeling as described in various publications [22, 23].The described late lumen enlargement in coronary arteries after balloon angioplasty with paclitaxel-coated balloon could lead to beneficial long-term results, specifically in SVD where vessel diameter is small. Sirolimus and its derivatives have also been evaluated as antiproliferative drug in DCB. Limitations of the “limus” substances are lower transfer rate of the drug compared to paclitaxel and the need for longer persistence in the tissue to develop the full antiproliferative effect [24]. Current data suggests, however, that some formulations seem to have at least a similar effect on the neointimal growth and therefore on late lumen loss [25, 26]. Thus, the subject of current research is to find the optimal formulation for “limus” substances to achieve meaningful clinical results.

**Table 1:** Drug-coated balloons currently available

*Name*	*Manufacturer*	*Dosage (μg/mm*^*2*^*)*	*Coating*	*Characteristics*	*Approval*
***Paclitaxel***					
*Agent*	Boston Scientific	2	Citrate ester excipient (acetyl tributyl citrate)	30 s inflation time	CE certified
*Danubio*	Minvasys	2.5	Butyryl-trihexyl citrate	30 s inflation time	CE certified
*Dior*	Eurocor	3	Aleuritic and shelloic acid (shellac coating)	20–30 s inflation time	
*Elutax-SV*	Aachen Resonance	2.2	No additives, two layers of paclitaxel	30–60 s inflation time; 10% remains on the balloon	CE certified
*Essential*	iVascular	3	Microcrystalline coating	30–60 s inflation time	CE certified
*Pantera Lux*	Biotronik	3	Butyryl-trihexyl citrate	30 s inflation time	CE certified
*Prevail DCB*	Medtronic	3.5	Urea		
*Protégé and Protégé NC*	Blue Medical	3	Drug component encapsulated in wings	30 s inflation time; also available as non-compliant balloon	CE certified
*RESTORE*	Cardionovum	3	Shellac	45 s inflation time	CE certified
*SeQuent Please Neo*	B Braun	3	Iopromide (hydrophilic spacer)	40 s inflation time; 4.5% remains on the balloon	CE certified
***Sirolimus***					
*Magic Touch*	Concept Medical	1.27	Phospholipid bi-layer	45 s inflation time	CE certified
*Sequent Please SCB*	B Braun	4	Crystalline sirolimus		CE certified
*Selution SLR*	Med Alliance		Micro-reservoirs made from biodegradable polymer		
*Virtue*	Orchestra Biomed		Nanoparticles made from biodegradable polyester-based polymer		

*CE*, Conformité Européenne; *FDA*, Food and Drug Administration of the United States of America

In order to achieve optimal results, a careful lesion preparation is key. It is therefore recommended to use a semi- or noncompliant balloon with a balloon-to-artery ratio of 1:1 or smaller and eventually specialist balloons or adjunctive methods such as rotablation prior to DCB use as described in current guidelines [20, 21]. It has been shown that optimal lesion preparation reduces event rates after DCB in patients with ISR [27], which is probably also transferable to the treatment in SVD. After the lesion preparation and prior to DCB delivery, the following points should be considered: maximum inflation of the balloon with the correct size for the treated vessel, residual stenosis of ≤ 30%, TIMI (Thrombolysis In Myocardial infarction) 3 and absence of dissections type C or higher (Fig. 1B); in case these conditions are not met, the implantation of a DES should be considered. When handling the DCB, care should be taken not to touch or allow the substance on the balloon to get wet, as this can already cause the drug to release. In addition, attention should be paid to the specific instructions of the respective balloon brand with regard to the transit time and the minimum inflation time for drug delivery. DCB should cover the prepared lesion and at least 2 mm proximally or distally to avoid geographical mismatch.

According to expert opinion [17], it is recommended to administer double platelet inhibition (DAPT) for only 1 month after the use of DCB. This short treatment time is an advantage for patients at increased risk of bleeding and has already shown good results in clinical trials in patients with stable SVD [14••, 28]. Given the very low acute vessel thrombosis risk [29], the duration of DAPT could be even further shortened in patients with very high bleeding risk; however, there is not enough data to support this notion. Current guidelines recommend DCB for patients with coronary ISR (class I, level of evidence A), but several non-randomized and randomized trials have shown sufficient efficacy and safety for the use in SVD. In addition, DCB are investigated as an attractive alternative in patients with bifurcation lesions and also de novo lesions in larger vessels.

### Drug-coated Balloons in Small Vessel Disease – Clinical Trials

#### Non-randomized Trials

In a retrospective study [30], 287 patients were treated either by 2 mm DCB (SeQuent Please, SeQuent Please neo and In.Pact Falcon; n = 87) or with a 2 mm second-generation DES (Xience Xpedition SV, Xience Alpine and Resolute Onyx; n = 200). In 7 patients with DCB bailout stenting was necessary. Stent thrombosis was seen in 4 patients (2.0%) with DES, but no vessel thrombosis was documented in the DCB group. Target lesion failure (TLF) at 12 months was similar between the groups (7.0% in DCB versus 8.2% DES; p = 0.73). Another retrospective study included 335 patients with [31], a vessel diameter of ≤ 2.5 mm receiving either DCB (SeQuent Please; n = 172) or a second-generation DES (Resolute Integrity, Xience, Promus Element, Biomatrix, Nobori; n = 163). Rates of bailout stenting were not reported. All patients received pre-dilation. There was no difference in MACE (11.6% versus 11.7%; p = 1.00) and no difference in TLR (5.2 versus 3.7, p = 0.601) between the two treatment groups after 12 months. In a large cohort of 7655 patients [29], 1197 patients received a DCB (SeQuent Please, In.Pact Falcon, Pantera Lux) and 6458 patients received a second or third-generation DES (Xience, Promus, Synergy, Resolute, Orsiro or Nobori). In 8% of lesions, bailout stenting was performed after DCB. After propensity score matching, DCB was associated with similar risk for target lesion revascularization (TLR; adjusted HR 1.05; 95% CI 0.72–1.53) but significantly lower risk for target lesion thrombosis (adjusted HR 0.18; 95% CI 0.04–0.82) compared to DES.

#### Randomized Controlled Trials

Randomized controlled trials comparing DES with DCB in de novo SVD are summarized in Table 2.

**Table 2:** Randomised controlled studies comparing newer-generation drug-eluting stent with drug-coated balloons in de novo small vessel disease

*Study*	*Device (n)*	*Vessel diameter — inclusion (mm)*	*Bailout Stenting (n)*	*DAPT DURATION (months)*	*Predilatation (n)*	*MACE*	*Outcome (DCB versus DES)*	*Follow-up (months)*
*PICCOLETO, 2010*	DCB: 28, Dior I (Paclitaxel) DES: 29, Taxus Liberté (Paclitaxel)	≤ 2.75	10 (BMS)	DCB: 1–3 DES: 12	DCB: 7 DES: 25	Death, MI, TLR	MACE (35.7% versus 13.8%, p = 0.054) TLR (32.1% versus 10.3%, p = 0.15)	9
*BELLO, 2012*	DCB: 90, Falcon (Paclitaxel) DES: 92, Taxus Liberté (Paclitaxel)	< 2.8	19 (BMS)	DCB: 2 DES: 12	DCB: 91 DES: 81	Death, MI, TLR	MACE (10% versus 16.3%, p = 0.21) TLR (4.4% versus 7.6%, p = 0.37)	6
*BASKET-SMALL 2, 2018*	DCB: 382, SequentPlease (Paclitaxel) DES: 376, Xience (Everolimus), Taxus (Paclitaxel)	2–3	19 (DES)	DCB: 1–12 DES: 6–12	DCB: 382 DES: 376	Cardiac death, MI, TLR	MACE (7.3% versus 7.5%, p = 0.92) TLR (3.4% versus 4.5%, p = 0.45)	12
*RESTORE SVD, 2018*	DCB: 116, SequentPlease, In.Pact Falcon, Pantera Lux, (Paclitaxel) DES: 114, Resolut Integrity(Zotarolimus)	2.25–2.75	6	> 6	DCB: 116 DES: 114	Death, MI, any revascularization	MACE (9.6% versus 9.6%, p = 1.0) TLF (4.4% versus 2.6%, p = 0.72)	9
*PICCOLETO II, 2020*	DCB: 118, Elutax SV (Paclitaxel) DES: 114, Xience EES (Everolimus)	2–2.75	8	DCB: 1 DES: 6–12	DCB: 99 DES: 78	Cardiac death, MI, TLR	MACE (5.6% versus 7.5%, p = 0.55) TLR (5.6% versus 5.6%, p = 0.8)	12

*BMS*, bare metal stent; *DCB*, drug-coated balloon; *DES*, drug-eluting stent; *MACE*, major cardiac adverse cardiac events; *MI*, myocardial infarction; *TLR*, target lesion revascularization; *TLF*, target lesion failure

In the PICCOLETO study (Paclitaxel-Coated Balloon Versus Drug-Eluting Stent During PCI of Small Coronary Vessels) [32], 57 patients with SVD (vessel diameter ≤ 2.75 mm) were randomized to either paclitaxel-coated balloon (n = 28, Dior I/Eucor) or first-generation paclitaxel-eluting stent (n = 29, Taxus/Boston Scientific). Due to higher rate of MACE (36% in DCB versus 14% in DES, p = 0.054) and higher target lesion stenosis (44% in DCB versus 24% in DES, p = 0.029) the study was prematurely interrupted after an interim analysis at 6 months. This result was due to the use of first-generation DCB that was providing a significantly lower drug concentration into the tissue due to its composition and therefore was less effective in the inhibition of neointimal proliferation.

The BELLO study (Balloon Elution and Late Loss Optimization) [33] randomized 182 patients with SVD (vessel diameter < 2.8 mm) to paclitaxel-coated balloon (n = 90, In.PACT Falcon) or paclitaxel-eluting stent (n = 92, Taxus Liberte/Boston Scientific). Bailout stenting was required in 20% of DCB and was performed using BMS. MACE (10% in DEB versus 16.3% in DES, p = 0.21), TLR (4.4% in DEB versus 7.6% in DES, p = 0.37) and angiographic restenosis (8.9% in DEB versus 14.1%, p = 0.25) was similar between DCB and DES after 6 months. The efficacy was confirmed up to 3 years [34]. In this study, there was a routine predilatation rate of 97% compared to only 25% in PICCOLETO.

In the randomized controlled BASKET-SMALL 2 trial (Basel Kosten-Effektivitäts trial, Drug-Coated Balloons for Small Coronary Artery Disease) [14••] 758 patients with SVD (vessel diameter < 3 mm) were treated with either a paclitaxel-coated balloon (n = 382, SeQuent Please) or a second-generation paclitaxel-eluting or everolimus-eluting DES (n = 376, Taxus Element or Xience/Abott Vascular). All patients received predilatation and were only randomized, if certain angiographic criteria were met (no high-grade dissection, no TIMI flow < 3 and residual stenosis ≤ 30%). After 12 months MACE (7.3% in DCB versus 7.5% in DES, p = 0.92) as well as its components (DCB versus DES: cardiac death 3.1% versus 1.3%, p = 0.11, non-fatal myocardial infarction (MI) 1.6% versus 3.5%, p = 0.11 and target vessel revascularization 3.4% versus 4.5%, p = 0.448) did not differ between DCB and DES, and non-inferiority of DCB vs. DES was established. In this trial, the efficacy and safety of DCB versus DES in the treatment of de novo SVD could be confirmed up to 3 years [15•].

In the RESTORE SVD trial (Drug-coated Balloon Versus Drug-Eluting Stent for Small-Vessel Disease) [35], 230 patients with SVD (vessel diameter ≥ 2.25 mm and vs. ≤ 2.75 mm) received either a paclitaxel-coated balloon (n = 116, Restore/Cardionovum) or a zotarolimus-eluting stent (n = 114, Resolute Integrity/Medtronic). At 9 months, similar in-segment percent diameter stenosis was found (29.6 ± 2% versus 24.1 ± 2%, p < 0.001 for non-inferiority) and there was no difference in TLR, cardiac death, MI and any revascularization. No difference was also found in TLF (4.4% versus 2.6%, p = 0.72) after 1 year and up to 2 years (5.2 versus 3.7%, p = 0.75) [36].

Finally, the PICCOLETO II (Drug Eluting Balloon Efficacy for Small Coronary Vessel Disease Treatment) trial [37•] randomized 232 patients with de novo SVD lesions to paclitaxel-coated balloon (n = 118, Elutax) or everolimus-eluting DES (n = 114, Xience). In-lesion late lumen loss was significantly lower in the DCB group (0.04 vs. 0.17 mm; p < 0.001 for noninferiority; p = 0.03 for superiority), but no difference was found in percent diameter stenosis and minimal lumen diameter after 6 months. After 12 months, there was no significant difference in MACE (7.5% in DES versus 5.6% in DCB, p = 0.55), spontaneous MI (4.7% in DES versus 1.9% in DCB; p = 0.23) and vessel thrombosis (1.8% in DES versus 0% in DCB, p = 0.15).

## Conclusion

The advantage of a DCB-only approach in comparison with DES is that nothing is left behind, but still a homogeneous distribution of antiproliferative drug ensures a good long-term result with a potential positive remodeling of the vessel and even enables a shorter duration of DAPT. Current data supports the use of DCB in SVD, as it has been shown that DCB compared to DES have a similar efficacy and safety. However, further large randomized studies with longer follow-up periods are needed, especially with the emerging “limus”-based DCB.
